# Perceived patient navigator services and characteristics to address barriers to linkage to hepatitis C care among people released from provincial prison in Quebec, Canada

**DOI:** 10.1016/j.drugpo.2024.104624

**Published:** 2024-10-18

**Authors:** Nadine Kronfli, Andrea Mambro, Lindsey R. Riback, David Ortiz-Paredes, Camille Dussault, Sylvie Chalifoux, Lina del Balso, Apostolia Petropoulos, Mona Lim, Alexandros Halavrezos, Giada Sebastiani, Marina B Klein, Bertrand Lebouche, Joseph Cox, Matthew J. Akiyama

**Affiliations:** a Department of Medicine, Division of Infectious Disease and Chronic Viral Illness Service, McGill University, Montreal, Quebec, Canada; b Centre for Outcomes Research and Evaluation, Research Institute of the McGill University Health Centre, Montreal, Quebec, Canada; c Montefiore Medical Center/Albert Einstein College of Medicine, New York City, NY, USA; d Department of Medicine, Division of Gastroenterology and Hepatology, McGill University, Montreal, Quebec, Canada; e Department of Family Medicine, McGill University, Montreal, Quebec, Canada; f Department of Epidemiology, Biostatistics and Occupational Health, McGill University, Montreal, Quebec, Canada

**Keywords:** Prison, Patient navigation, Hepatitis C, Barrier, Facilitator, Socio-ecological model, Patient navigator

## Abstract

**Background:**

Patient navigation increases linkage to hepatitis C virus (HCV) care following release from prison; however, little is known about the services patient navigators should provide to maximize linkage to care. We aimed to identify perceived barriers and facilitators to linkage to HCV care post-release, and to determine patient navigator services and characteristics best suited to address barriers to linkage to care among people released from prison.

**Methods:**

Ten semi-structured interviews were conducted with adult (age ≥18 years) men living with chronic HCV, released from the largest Quebec provincial prison, and linked to HCV care by a patient navigator. Interviews were guided by the Socio-Ecological Model (SEM) and aimed to explore the multi-level barriers and facilitators to linkage to HCV care post-release. Interviews were audio-recorded, transcribed, and analyzed using a deductive, thematic approach.

**Results:**

The median age of participants was 54 years. Barriers to linkage to HCV care included competing priorities post-release (e.g., substance use, mental health issues, unstable housing), stigma (related to HCV, injection drug use, and incarceration), and lack of transportation. Facilitators included social support, established relationships with existing healthcare providers, prior cure with direct-acting antivirals, and HCV-related health literacy and knowledge. Perceived essential patient navigator services to enhance linkage included pre-release discharge appointments, housing assistance, and facilitated transportation to HCV appointments. Ensuring a consistent, non-judgemental, and empathetic patient navigator were considered important characteristics; lived experiences of incarceration and/or HCV were not felt to be essential for a patient navigator.

**Conclusions:**

Interventions that seek to improve linkage to HCV care for people following release from prison should address many levels (individual, interpersonal, and policy) of the SEM. While people experience several competing priorities post-release, having an empathetic and consistent patient navigator, regardless of their lived experiences of HCV and/or incarceration, may improve linkage to HCV care post-release.

## Introduction

An estimated 11.5 million people are incarcerated daily with over 30 million cycling through prison annually, a number that is growing in part due to the criminalisation of drug use (Institute for Crime & Justice Policy Research, 2022; Penal Reform International, 2023). Over half of people who inject drugs experience incarceration (Degenhardt et al., 2023). This, coupled with high turnover and recidivism rates, point to the urgent need to prioritize prisons as settings for the treatment of people living with hepatitis C virus (HCV) to meet the World Health Organization’s (WHO) global HCV elimination goals by 2030 (WHO, 2016, 2022a; Akiyama et al., 2021, 2022; Rockstroh et al., 2023). However, despite the availability of prison-based HCV treatment programs, short sentences and a lack of continuity of care programs following release mean that many people with chronic HCV are released into the community untreated (Kronfli et al., 2021, 2019a, 2019b; Kronfli & Cox, 2018).

Individuals face several competing priorities post-release which contribute to markedly high rates of death immediately following community re-integration (Borschmann et al., 2024; Binswanger et al., 2007, 2011) and reduced likelihood for linkage to health care, including HCV (Kronfli et al., 2019a; Papaluca et al., 2019; Cocoros et al., 2014; Beckwith et al., 2016). There is a thorough understanding of the barriers to linkage to HCV care, which include unstable housing, poor social support, experiences of stigma and discrimination in health care settings, and continuation or resumption of substance use (Yanes-Lane et al., 2020; Porter et al., 2023; Kamat et al., 2023; Cepeda et al., 2015). Few facilitators to linkage to HCV care post-release have been identified in real-world studies (Akiyama et al., 2019; Papaluca et al., 2022). These include discharge planning, social support, having an existing primary care provider, receipt of opioid agonist therapy, and successful contact following release (Akiyama et al., 2019; Papaluca et al., 2022). While some research exists to guide the development of novel linkage to HCV care strategies and programs, much of the research has thus far focused on optimizing linkage to HIV care post-release from prison. To design, implement, and evaluate interventions that seek to improve linkage to HCV care following release, a thorough understanding of the barriers and facilitators to care is needed. While some barriers and facilitators to HCV care post-release are expected to overlap, geographic and setting specific differences underscore the importance of understanding local contexts (Kamat et al., 2023; Akiyama et al., 2020; Crowley et al., 2018, 2019).

Patient navigation has been shown to increase linkage to HCV care following release from a carceral setting (Cabezas et al., 2021; Akiyama et al., 2019; Papaluca et al., 2022). Patient navigation is designed to support individuals in finding their way through health and social care systems, and to assist individuals with overcoming barriers to accessing services (Budde et al., 2022). Based on our recent systematic review (Yanes-Lane et al., 2020), a multidisciplinary care team consisting of a nurse, social worker, and patient navigator may be best positioned to address the most common barriers to linkage to HCV care following release from prison. While nurses and social workers typically perform well-defined tasks, key roles of patient navigators often depend on their skills and experience (Budde et al., 2022). To date, very little is known about the services that patient navigators should provide following release to maximize the success for linkage. Little is also known about patient navigator characteristics to optimize linkage to care. WHO recently recommended *peer* navigators to support the engagement of key populations in viral hepatitis care, suggesting that patient navigators should have some form of lived experience such as incarceration and/or HCV (WHO, 2022a). Given these knowledge gaps, we aimed to identify barriers and facilitators to linkage to HCV care among people released from the largest Quebec provincial prison while engaged with a multidisciplinary care team, and to determine patient navigator services and characteristics best suited to address barriers to HCV care post-release.

## Methods

### Study design

We conducted a theory-informed qualitative study using semi-structured in-person interviews. People living with chronic HCV and released from prison were interviewed to identify both barriers and facilitators to linkage to HCV care, as well as patient navigator services and characteristics best suited to address barriers to linkage to HCV care post-release.

### Setting and participants

There are 16 provincial prisons in Quebec, housing approximately 4442 incarcerated individuals in 2022/2023 (Statistics Canada, 2024). L’Établissement de détention de Montréal (EDM) is the largest provincial prison in Quebec, with a capacity of 1400 adult men (≥18 years old) (Ministère de la Sécurité Publique du Québec, 2019). In Canada, individuals awaiting sentencing or who are sentenced for less than two years are housed in provincial prisons while individuals sentenced for two years or more are housed in federal prisons (Kronfli & Cox, 2018; Kronfli et al., 2019b, 2021). The mean duration of incarceration in Canadian provincial prisons is 28 days (Malakieh, 2020).

Individuals were recruited after enrolling in a larger study (‘Beyond Prison Walls’) that aimed to evaluate the impact of a multidisciplinary care team, consisting of a nurse, social worker, and patient navigator, on linkage to HCV care among individuals with chronic HCV following release from prison. In Quebec provincial prisons, incarceration length is an important barrier to the initiation of HCV treatment during incarceration (Kronfli et al., 2021). Beyond Prison Walls sought to recruit individuals aged 18 years or older and serving a sentence of 2–12 weeks—considered the minimum duration required to undergo a diagnostic work-up for HCV but insufficiently long to complete treatment while incarcerated following the necessary approvals. In this qualitative sub-study, all participants who were linked to HCV care with the patient navigator were included. Participants were excluded if they posed a security risk to the patient navigator. Each participant with confirmed chronic HCV met with a nurse, who provided education on HCV and its treatment, a social worker, who conducted a harm reduction needs assessment and provided community referrals at the time of release to address important social determinants of health, and a patient navigator, who scheduled an HCV care appointment for participants prior to release. The entire multidisciplinary care team was based at EDM. The patient navigator was a trained research assistant with expertise in qualitative research in medical anthropology. The patient navigator (AM) was not a peer and did not have lived experiences of HCV or incarceration. The patient navigator met with each participant at least twice before their release. Following community re-entry, the patient navigator also made reminder phone calls to participants prior to their HCV care appointments, met participants at designated locations prior to linkage, accompanied (or “navigated”) participants to their HCV care appointment if desired by participants, and rescheduled appointments if needed.

### Guiding framework: the socio-ecological model

We adopted the Socio-Ecological Model (SEM) of McLeroy et al. (1988) to understand barriers and facilitators to linkage to HCV care across several levels. Socioecological systems theory demonstrates the nested and interdependent nature of human development and interactions at the personal, relational, and collective levels. As such, the SEM was developed recognizing that individuals affect and are affected by a complex range of social influences and environmental interactions. The SEM involves five levels of influence: individual, interpersonal, organizational, community, and policy (Michaels et al., 2022). Each level represents a distinct sphere of influence, although they are all interconnected and reflect how changes or interventions at one level can impact the others. This comprehensive framework helps in understanding and addressing the multifaceted determinants of linkage to care post-release, facilitating targeted and effective interventions across various contexts.

### Interview guide development

The interview guide was developed by the study team using the SEM across five levels. The different levels of the SEM are presented in Table 1, with their definitions and examples from the interview guide. The interview guide included one to five questions for each of the SEM levels. Given anticipated challenges with the recruitment of participants due to potential competing priorities post-release, the interview guide was not pilot tested. Instead, we proceeded to collect data from the study’s outset.

### Data collection

Participants who were successfully linked to an HCV care provider post-release were recruited by the patient navigator into this qualitative sub-study directly following their HCV appointments. Participants provided written informed consent prior to participation. Interviews were conducted in a public location (e.g., coffee shop, hospital cafeteria, park) by the patient navigator, who ensured privacy by choosing a quiet, secluded area and maintaining a respectful distance from others.

All interviews were conducted in person by one of two members of the research team (AM and DOP) in English or French. All interviews were audio-recorded, and recordings were transcribed verbatim, translated to English by a professional translator, and anonymized simultaneously. Transcripts were not revised or verified by participants due to challenges in maintaining contact, but were verified for accuracy by AM. All interviews were included in the analysis.

Data saturation in qualitative research is an important indicator of quality and trustworthiness. In this study, we gauged saturation by whether new themes emerged after each interview. To ensure that we achieved an adequate sample for content validity, we used the 10 + 3 decision rule (Francis et al., 2009), whereby we aimed to conduct 10 initial interviews, followed by an additional three interviews if data saturation was not achieved and until no new themes emerged. If data saturation was reached after 10 interviews, additional interviews were not required.

Participants received $50 CAD for their study participation (Mambro et al., 2024a). This was provided in cash, as bus transportation assistance, or a gift card. This study was approved by the McGill University Health Centre Research Ethics Board (#2020-6168).

### Data analysis

All transcripts were independently double-coded using Dedoose software (version 9) by two members of the research team trained in qualitative research (AM and LRR). Coders met after coding the first four transcripts to discuss and resolve any discrepancies and to develop a uniform coding scheme. A deductive thematic analysis was performed stratified by SEM levels. Barriers and facilitators were categorized according to their respective SEM levels, and the analysis focused on understanding how these barriers and facilitators interacted to influence outcomes. Themes were discussed with two additional members of the research team (NK and MJA). Discrepancies were resolved via consensus. Our findings are reported in line with the Consolidated criteria for Reporting Qualitative research (COREQ) guidelines (Tong et al., 2006), which can be found in Supplementary material.

## Results

From January 10, 2020 to April 28, 2024, 20 people with chronic HCV were released from EDM, among whom 16 (80 %) were linked to care between February 18, 2020 and July 7, 2024. Among these, 12 (75 %) desired patient navigation to their HCV care appointment. Of the 12, 10 consented to study participation; two refused due to competing priorities post-appointment. These interviews were conducted between November 11, 2022 and July 7, 2024. All 10 participants self-identified as men. The median age of participants was 54 years old (Table 2). All but one participant was interviewed immediately following his linkage to care appointment. Interviews lasted an average of 50 min (range: 45–90 min).

### Barriers and facilitators to linkage to HCV care post-release using the SEM model

#### Individual

##### Health literacy, knowledge, and attitudes toward HCV treatment

Many participants discussed how they obtained information about HCV, and how their understanding of this information, in addition to their pre-existing knowledge of the disease, its transmission, treatment options, and their perceptions or beliefs impacted the choices they made about engaging in HCV care. For most participants, information and support from healthcare providers helped improve their health literacy and acted as facilitators to linkage to HCV care.

Overall, most participants had a basic understanding of the virus, its modes of transmission, and long-term consequences. Most participants demonstrated an understanding of HCV as a virus which affected the liver and which was primarily transmitted through blood, leading to potentially severe outcomes such as cirrhosis if untreated. This basic understanding better equipped participants to link to HCV care post-release.

“It’s a liver disease. It’s a virus that attacks the liver and that is only caught by blood. And the hepatitis C virus, well, that’s it, you end up by having cirrhosis and then, that’s it. It’s bad. And then, it’s sexually transmittable but more so by blood.”—P2

One participant, who was screened for HCV during his incarceration, mentioned that the information he received about HCV influenced his decision to seek care. This participant learned about the possibility of cure through his engagement with the research nurse.

“It’s through a questionnaire. I learned this through a questionnaire. That’s how I knew! (laughs) … It was the nurse who came to [the prison] to see me and who told me that, you know, roughly about it, and I answered a questionnaire too. They were explaining it to me as they went along, you know. They asked me “If it was curable,” and I said, “Surely hepatitis C can be cured these days.” I knew it, I knew it was curable, you know?”—P1

Other participants highlighted the importance of educational material provided by venues commonly frequented following release from prison including shelters. These resources contributed to their understanding of HCV and served to motivate individuals to engage in care.

“I’ve also relied on informational pamphlets and materials provided by clinics and shelters. Learning about the potential consequences of untreated hepatitis C and the availability to get treated. Knowing that there are options available and that I don’t have to face this for a long, long time makes me feel like I have more time. It motivated me to go through all the steps.”—P9

Several participants highlighted the difficulties in accessing information about HCV and HCV care due to their specific circumstances such as lack of Internet access or limited health literacy. Complex medical terminology and a lack of easy-to-understand resources made it challenging to understand essential information about their infection and the available treatment options. One participant shared,

“Accessing information on HCV and HCV care has been a bit challenging given my circumstances. I don’t have regular access to the Internet or reliable sources of information.”—P1

Other participants illustrated how they sought their own resources and services once diagnosed to expand their knowledge, which they subsequently used to make informed decisions about seeking care. This newly acquired knowledge empowered them to take proactive steps, including seeking hepatitis A and B vaccination, and acted as a facilitator to linkage to HCV care.

“And then I did some research on the Internet… That’s when I found out that there was no vaccine for hepatitis C. There was a vaccine for hepatitis B, but there was no vaccine for hepatitis C. There was a vaccine for hepatitis A, hepatitis B, which I was given … and I think it was you [the patient navigator] who arranged the appointments. So, I have the vaccines for hepatitis A and hepatitis B, and I saw on the Internet that sometimes, when you’re infected with hepatitis C, sometimes it goes away on its own, but other times, you really have to undergo treatment. And I know that in the past, treatment was for a year, wasn’t it? And I also know that there was a 50 % chance of getting rid of it, whereas the new treatment lasts 8 weeks, and I think there’s more than a 90 % chance of getting rid of it. That’s it, right? And I also know that hepatitis C causes cirrhosis of the liver in the long term.”—P2

“I’ve made it a point to ask questions and seek guidance from healthcare, outreach workers, etc.”—P10

Many participants highlighted the severe consequences of untreated HCV and how having learned that HCV is curable provided relief and motivation to engage in the treatment process.

“Because I know that it’s a disease that kills, and I know that I can be cured, so it would be stupid not to try.”—P7

Overall, information sought from various sources served to improve participants’ health literacy and knowledge, and subsequently influence their attitudes toward HCV treatment. However, linkage to HCV care was often compromised for those without similar access to resources.

#### Motivation for HCV treatment

Initially, some participants lacked motivation to link to HCV care as they attributed their symptoms to other factors, including harsh living conditions, leading to delays in seeking medical care. Shifts in motivation to seek care often occurred when their health had significantly deteriorated. For instance,

“I’ve been feeling very unwell over the past few months, feeling tired, abdominal pain, and a general sense of malaise. Initially, I brushed it off, figured it was because of my conditions of living on the streets. But, as time passed, this feeling didn’t stop and even worsened. I thought this was going to be the rest of my life. You know? I started to think something was wrong, but I didn’t want to tell anybody. It set off alarm bells in my mind.”—P9

All participants revealed common themes that facilitated their inner drive and willingness to initiate and adhere to HCV treatment. All participants were highly motivated to improve their health and well-being. For example,

“I knew that seeking hepatitis C care was essential not only for my own well-being but also to break the cycle of illness and pain that had plagued me for so long.”—P9

Getting treated for hepatitis C was part of me changing my life, leaving behind what I used to do, and starting fresh.”—P7

“I wanted to show myself and others that I could turn things around. Getting rid of this disease was one of those steps.”—P5

The need to be “clean” to prevent transmitting the virus motivated participants to seek treatment. Participants were thus able to see the value of treatment beyond their personal health.

“I want to be clean, because I don’t want to hurt anyone. I don’t want to contaminate anyone.”—P8

A strong personal motivation to improve health and avoid complications was evident among most participants. This motivation drove them to engage in health care services and initiate treatment.

“Well, to get better, you know, to cure my virus. To get through it and avoid liver problems.”—P10

Other participants highlighted a new commitment to self-care.

“I was followed by a nurse. It really was okay, we had a relationship, an exchange, I did my treatment, and I motivated myself to do my treatment, and I did it. My resolution for my thirties is that I want to take care of myself.”—P8, interviewed after treatment completion

Overall, participants were motivated to seek HCV treatment for various reasons including to improve their health, prevent transmission, and overcome their past—as it relates to breaking free from repetitive cycles of illness, pain, and substance use.

##### Lived experiences, intersecting stigma and social isolation, and competing priorities preventing linkage to HCV care

Participants discussed personal experiences such as substance use, mental health issues, stigma, and social isolation as barriers to seeking HCV care. These intersecting experiences often heightened the challenges in accessing health care. For instance, untreated mental health issues, like bipolar disorder, often led to impulsive actions that resulted in reincarceration, complicating efforts to seek health care post-release. Similarly, stigma around homelessness, HCV, and mental illness compounded the isolation participants felt, creating a cycle of avoidance in accessing care. One participant explained his frustration with his overall lack of support around bipolar disorder, which in turn complicated his ability to seek appropriate care.

“Well, I have bipolar disorder … I need help sometimes and get frustrated when nobody doesn’t help me. I can’t help it. I feel like nobody is listening to me. And then I get impulsive and destructive. And that’s why I got back in there [prison].”—P9

Participants reported instances in which these lived experiences intersected with one another. For example, intersecting stigma and social isolation associated with HCV, homelessness, and mental illness created significant barriers to linkage to care. A participant shared,

“Yeah, I did hesitate, to be honest. Living on the streets with hepatitis C and struggling with inside my head, it’s easy to feel overwhelmed and lost in the shuffle. There were times when I questioned whether I was worthy of receiving care or if I even deserved to get better. The stigma surrounding homelessness and mental illness can be incredibly isolating, and it made me doubt whether anyone would take me seriously or offer me the help I needed. Plus, the thought of facing my diagnosis head-on was terrifying. I was scared of what the future held and whether I’d be able to handle the treatment process. But ultimately, I knew that I couldn’t continue living in denial. I had to confront my illness and take the necessary steps to get better, for my own sake and for the sake of those who cared about me.”—P9

Almost all participants had competing priorities following release from prison that made it challenging to link to their HCV care appointments. This included reconnecting with their social networks.

“Well, it’s because I was happy to be out, and I was just seeing my friend. He … we had a lot to talk about. But you know, like this morning, I saw him again. I said, “No, no! There are things I need to do around here” I knew that I wasn’t happy that I missed [my appointment] the first time, you know? I regretted it.”—P6

However, participants found themselves withdrawing during low points, ignoring calls from friends. This deliberate self-isolation stemmed from a reluctance to burden others, which led to further isolation.

“If I want to live 10 more years, I have to help myself. I have to help myself a whole lot, because sometimes I let my chin go. You know, like, last week, I know she [patient navigator] tried to call me for two days, but I didn’t even answer the phone. She called my friend to check on me, but, you know, I wasn’t feeling well. You know, when I’m not all right, I don’t see anyone, I stay by myself. That’s my way of being, I don’t want to bother people. There’s no point in bothering people.”—P1

Ensuring stable housing following release from prison was the most commonly reported competing priority for participants. Participants explained the broader challenges encountered when transitioning from incarceration to homelessness, as well as the complexities of managing multiple health conditions without stable housing.

“Transitioning from life in prison to life on the streets was like stepping into a whole other world. Suddenly, I was faced with a whole new set of challenges and barriers to accessing care. From finding a stable place to sleep to figuring out where to get my next meal, just getting through each day was a struggle.”—P9

“I don’t have a place to live, you know, I’m going to have treatments for my cancer, have treatments for hepatitis … No place to stay, things are bad, you know, right?”—P1

One participant described the difficulties faced when unstable housing is combined with non-existent means of communication:

“Without a permanent address or reliable means of communication, it felt like I was constantly hitting dead ends and running in circles.”—P9

Conversely, prior and positive lived experience of HCV treatment acted as facilitators to linkage to HCV care. Participants who had previously undergone HCV treatment and experienced positive outcomes were more likely to seek treatment when re-infected, highlighting the importance of successful health care interactions.

“Since I’ve already done the treatment, it’s not something I’m afraid of … I wasn’t too upset, I knew that it was not unpleasant, that I had to follow the treatments.”—P3

“As someone who has been through it myself, I know how big of a responsibility it is to keep going to health care appointments. And most importantly, don’t be afraid to ask for help. There are people out there who care about your health and well-being and who are willing to support you every step of the way.”—P9

### Interpersonal

#### Communication and relationship building between patient and healthcare providers in facilitating linkage to care

Participants discussed their ability to communicate as patients with healthcare providers through personalized support, clear information sharing, emotional reassurance, regular follow-ups that encouraged proactive engagement, fostered trust and familiarity, and ultimately facilitated linkage to care. This helped address participants’ fears and optimize adherence to treatment following successful linkage to care.

Some, who were aware of their infection prior to being tested, were reluctant to engage in honest communication with the multidisciplinary care team about their diagnosis, often due to fear of stigma or potential consequences. Few participants described their intense fear of medical professionals.

“Honestly, I was still afraid to tell the nurse at prison at the beginning that I already had hepatitis C. Once she did my bloods though, it was helpful. She listened to what I was facing and asked what I wanted.”—P9

Others felt comfortable with the multidisciplinary care team immediately:

“When it comes to my health, I know I can ask the nurse for help. She gives me straight answers, and I don’t have to worry about getting bad advice.”—P5

“I talked more to the social worker. It was her who explained to me how treatment worked, and the nurse, as I told you, she met me 2–3 weeks before I was released, and she was astonished that they didn’t see me earlier, before. You know, she was surprised to see me so late, she didn’t understand why I hadn’t been taken care of before I got out, 2–3 weeks before getting out.”—P3

Most often, participants also highlighted their reliance on familiarity and comfort with specific healthcare providers as being a critical facilitator to linkage to care, as it directly influenced their willingness and ability to adhere to their treatment. Participants expressed a preference to continue care at their local health care facility due to established relationships with their existing nurse and/or doctor. The familiarity with these providers fostered a sense of comfort and trust.

“This is my neighbourhood, you know? This is really my place! You know, like I said ‘I might be transferred to [a hospital in Montreal], but that doesn’t appeal to me because I’m used to this nurse and this doctor,’ you know?”—P1

#### Social support in facilitating HCV care post-release

Almost all participants highlighted how their inter-personal relationships with friends, family, and peers contributed to their well-being and assisted with linkage to HCV care post-release. Most participants expressed that support from family members and friends was caring and non-judgemental.

“I told my dad that I have hepatitis C when he picked me up from prison. He is the reason I am close to [a city in Quebec] now. He was supporting me. When he learned that I was taking steps to seek hepatitis C care, his reactions were positive. He expressed genuine concern for me.”—P9

“My mother knew that I was sick. You know, and … Well, I took the first steps, I went to get treated.”—P8

Additionally, friends’ encouragement and shared experience provided reassurance about HCV.

“Yes, my friends. Yes, yes, they know. They reassured me. There was one who told me that it could be cured … He said, ‘It’s cured today, don’t worry!’ He used to work on, uh … at a [community organization]. He was giving out, you know, syringes, all kinds of things in the street, you know? Well, he worked at a [community organization]and he had it. He told me that it can be cured.”—P1

Direct referrals to healthcare providers by trusted friends and peers facilitated linkage to HCV care for some.

“They were people in the [therapy program], two of my friends were there, and they talked to me about it. One of them put me in touch with [name of nurse]. I called her, then … I just made an appointment with her, and that’s how it started.”—P7

Overall, social support played a key role in facilitating linkage to HCV care following release from prison, providing the needed encouragement and shared experiences.

### Organizational

#### Accessibility of healthcare providers and transportation challenges preventing linkage to care

Participants discussed difficulties in accessing health care services prior to engagement with the multidisciplinary care team, highlighting the inefficiencies in HCV treatment initiation that further complicated their ability to receive timely care. Many identified several barriers to linkage to care including accessibility and travel difficulties and a lack of follow-up and coordination. Collectively, they contributed to delays in receiving HCV care. Accessibility issues emerged as an important barrier as many participants had to travel considerable distances to their pre-assigned healthcare provider (i.e., primary care physician) or HCV care specialist.

“I had to travel all the way to [a municipality in Quebec]. I have no car, and I don’t live in [this municipality], never have lived there. She [the family doctor] was hard to access.”—P9

“But it was particularly difficult to get to the specialist, to [a municipality in Quebec], because I don’t live nearby.”—P2

Some participants, who had been reincarcerated, reported a lack of follow-up and coordination from prison-based healthcare providers. This lack of continuity of care acted as a barrier to ongoing HCV care engagement.

“Yes, because actually they started my treatment at [city in Quebec]. That was the first time I was on a program like that. And when I got transferred to [a prison in Montreal], they cut my medication, so the nurse said, “If you want your prescription back, you have to call a [community organization].”—P4

Participants also reported a lack of care continuity following treatment initiation and unsuccessful completion in the community.

“Since I left [the doctor], after that, it was difficult to be able to restart the treatment, and that’s what I found most difficult, and I don’t know about all that, so I didn’t know where to go, I didn’t know who to call.”—P4

At an organizational-level, participants experienced difficulties with accessing healthcare providers due to long distances and absent means of transportation, compromising linkage to HCV care. When engaged in HCV care, continuity of care was also challenging due to re-incarceration and lack of follow-up and coordination with prescribing physicians.

### Community

#### Navigating HCV information and care from community-based organizations

Following release, many individuals face significant challenges with their reintegration back into community. These often include barriers to accessing health care services, housing, and social support. Community-based organizations (CBOs) play a critical role in addressing these challenges by providing a wide range of services tailored to individuals’ needs. Post-release, participants often accessed CBOs that included crisis prevention centres. These centres provide immediate support and interventions to individuals in distress, including mental health services, substance use counseling, and emergency shelters. Other CBOs focus on long-term reintegration, offering services such as social work support, legal assistance, housing, and health care access. CBOs are particularly valuable in circumventing barriers such as stigma, transportations issues, and lack of trust in formal health care systems.

Participants noted that structured programs within CBOs, particularly those promoting peer interaction and open communication, played an important role in their reintegration by creating a sense of community and mutual support. One participant shared his positive experience with a crisis prevention program within a homeless shelter, highlighting the program’s supportive environment and the value of peer connections.

“[I]t was the program here at [homeless shelter], the [crisis prevention program], and I knew people who were following the program. In the program, we’d go out and sit in parks, and I’m not afraid to ask questions.”—P5

Participants emphasized the importance of CBOs in assisting with their reintegration into society following release from prison. The services provided by CBOs were often preferred to those provided by shelters. With this in mind, one participant emphasized the importance of enhancing community-based health care services in reaching underserved communities and addressing safety concerns of shelters.

“One thing I would say is to increase the availability of mobile health care units that can reach communities that have less opportunity. I don’t like downtown Montreal, but I did like mobile clinics that you can call and they come help you. The shelters aren’t safe places, we need to fix that.”—P4

An additional hypothetical facilitator to linkage to HCV care following release was the potential implementation of decentralized and on-site services and support programs within community settings such as shelters to resolve transportation and communication concerns. These services could be supported by peers with lived experience of HCV or homelessness, making the experience more relatable, thereby potentially enhancing engagement in care, particularly for marginalized populations such as those experiencing homelessness.

“If the shelters also had on-site testing, education, and treatment for hepatitis C, removing some of the barriers with transportation and communication. Also, setting up support programs where people who already had HCV help other people who are diagnosed and who have experienced homelessness themselves can serve as mentors.”—P9

Overall, CBOs were felt to be essential in supporting individuals’ reintegration into society after release from prison, offering vital services such as crisis prevention, health care access, and peer support. Participants preferred these services over those provided by shelters, citing the personal connections, familiarity, and safer environments offered by CBOs. They also advocated for expanding decentralized and mobile healthcare services in underserved areas to improve accessibility and address safety concerns. Integrating on-site health services and peer support within community settings could further enhance engagement with care, particularly for those facing challenges like homelessness or chronic hepatitis C infection.

### Policy

#### System-level barriers in accessing HCV care

In Quebec, the management of HCV care is hindered by several system-level barriers that delay access to care. For many, regulatory barriers such as requiring a valid health care card presented additional obstacles to linkage to HCV care post-release. Many individuals with expired health care cards required assistance to navigate the complex health care system to reactivate their cards for the purpose of receiving care post-release, a process that could take weeks to accomplish.

“I got my [health care] card in prison, you set that up for me because I lost my other one … I had already called the [provincial health care plan] to sign up for prescription insurance. But social assistance doesn’t know about it … I asked someone in social assistance if I can sign the papers to get my cheque. [They said] “It doesn’t work like that!” They told me I had to wait another 15 days to get my social assistance and start my medication. It’s frustrating!”—P5

As previously mentioned, securing stable housing was a predominant competing priority following release from prison. Several participants highlighted the need for supportive housing programs, funded at the provincial and national levels, tailored to people experiencing homelessness and health conditions like HCV as essential for improving health outcomes. Additional funding for wrap-around services, such as mental health and substance abuse programs, was reported to be important to address the root cause of homelessness.

“One major barrier that needs to be addressed is the lack of stable housing. Without a permanent address, it’s incredibly difficult to access health care services and continue in treatment. I would suggest expanding affordable housing initiatives and supportive housing programs that target people experiencing homelessness and health conditions like hepatitis C. I know after I complete this treatment and I am no longer eligible to stay at the [crisis prevention centre], I will be back on the streets. They try to help you to find an apartment, but the waiting lists are long. I’ll be back on the streets or back in prison. Providing stable housing not only improves health outcomes but also reduces the burden on emergency services and the criminal justice system. And we need more money, more funding for mental health services and substance abuse treatment programs would help address the issues that lead to homelessness.”—P9

Overall, several system-level barriers presented challenges in accessing HCV care for participants post-release, many of which could be resolved with increased funding and the addition of wrap-around services.

### Patient navigator services and characteristics best suited to address barriers to HCV care post-release

Although participants’ relationships with the patient navigator could have been included at the interpersonal level, we chose to present these results separately as it was a distinct study objective. In the evolving landscape of health care, the role of the patient navigator was highlighted among all participants as being critical to linkage to HCV care post-release serving as a link between healthcare providers in a complex system and individuals re-entering the community, thereby ensuring continuity of care and improving treatment outcomes.

“Maybe I wouldn’t have gone. Because … I wouldn’t care, you know? But at least you [the patient navigator] came, it encouraged me, and I was happy.”—P6

“It’s the approach, and what you [the patient navigator] offered as treatment, that’s what I liked. That’s why no damn person will replace you, right?”—P3

“Well, I’m going to follow you [the patient navigator], because I don’t know anything. I have trouble writing and reading, so I need an advocate.”—P6

“We’re talking about 10 days here. It’s been 10 days for me, I’ve been on edge. I’m scared shitless of doctors, damn it! Right? It’s because you’re [the patient navigator] here with me that I’m still here, because otherwise, damn it, I would have fucking quit a long time ago. Right? I would have just fucking left.”—P3

Participants identified key aspects of the patient navigator that improved their desire to link to HCV care following release including transportation and housing assistance, and referrals to therapy programs. Several participants pointed to the importance of the pre-release discharge appointments, organized by the patient navigator, as a facilitator to HCV care following release.

“I appreciate that you [the patient navigator] helped set up my appointments, making sure I didn’t fall through the cracks. I also leaned on you for support.”—P8

“When you [the patient navigator] came to see me at [the prison], you would tell me “such and such a date,” because you had told me in advance “such and such a date,” I had that.”—P2

“You [the patient navigator] took the lead. Then, I’m not even tempted to make the appointment, you know, it’s fatigue, too, that sets in. Anxiety, you know? Sometimes I don’t feel like doing anything anymore. For me, that’s what … when I no longer feel like doing anything, I don’t do anything! Well, I shouldn’t do that, you know. I know I shouldn’t. There, I know that when you handled my business, it helped me.”—P1

All participants highlighted that the patient navigator helped overcome transportation concerns by providing accompanied transportation assistance to and from appointments, which helped alleviate the emotional stress associated with navigating a complex health care system.

“It was particularly difficult to get to the specialist, to [a municipality in Quebec], because I don’t live nearby, but you [the patient navigator] helped me with a cab.”—P2

“The truth is that you [the patient navigator] helped me immensely, because I found myself in my father’s house, who had passed away, I had no means of transportation, and then you took care via the community centre, to get me to the clinic, and without you, without you, I think I wouldn’t be treated yet.”—P2

Interestingly, while housing assistance and referrals to treatment programs were not part of the patient navigator’s envisaged services in Beyond Prison Walls, oftentimes, they were requested by participants to help to alleviate important post-release competing priorities.

“Currently, I am living at a [crisis prevention centre] in a [municipality in Quebec] that you guys organized for me. I am forever grateful that I was able to go back there after they kicked me out in the past.”—P8

“I really need a form for an apartment. You know, next week, you’re [the patient navigator] going to come back and see me again to fill out (the form) for [low-income housing].”—P1

Finally, patient navigator services that began during incarceration and continued immediately following prison release were considered critical for linkage to HCV care.

“With you [the patient navigator], it was very easy. There was no waiting. You took care of me as soon as I got out of prison. So, I couldn’t ask for anything better.”—P2

Participants highlighted several characteristics of an “ideal” patient navigator which served to facilitate linkage to HCV care, including consistency. Notably, the provision of post-release care by the same individual was appreciated by all participants; having a single point of contact (i.e., one dedicated patient navigator) was a facilitator to linkage to care.

“You [the patient navigator] took me in, you offered me tests, you followed me from beginning to end. I owe it all to you.”—P2

“As someone who was struggling with mental illness and homelessness, I often felt stressed and unsure of where to turn for help. Having someone who could have reached out to me, provided information about available resources, and helped me with everything in the process of accessing care after prison would have been helpful. Having one person to turn to instead of many different people. I appreciate that the patient navigator had been able to accompany me to appointments and provide emotional support, it made the experience feel less long. But I also felt like I didn’t need to listen to anything the doctor was saying because you [the patient navigator] would explain it to me. So, it was less stressful. Having a dedicated patient navigator who could guide me through the process and offer ongoing support made a world of difference in my journey towards getting hepatitis C care.”—P9

Almost all participants illustrated how the patient navigator fostered a relationship based on trust, empathy, and free from judgement in the process of linkage to care. Participants expressed gratitude for the comprehensive and reliable care they received by the patient navigator. Participants felt heard and found their relationship with the patient navigator particularly nurturing, in contrast with the punitive carceral environments from which they emerged and where power dynamics are evident.

“You [the patient navigator] listened to what I was facing and asked what I wanted. You went out of your way to help me.”—P9

“You [the patient navigator] were forthcoming, your approach. I didn’t feel judged, let’s say. I don’t know how to put it, it’s not for nothing that you’re here.”—P3

“You [the patient navigator] are more professional. You don’t have a motive. The most important thing is that the patient navigator is empathetic, knowledgeable, and dedicated to supporting people like me.”—P9

When specifically asked about the importance of lived experiences of HCV and/or incarceration, only two participants highlighted that a patient navigator with such experience may help in building rapport and a trusting relationship, helping others to link to care following release.

“I think having a patient navigator who has been through a similar situation would be incredibly beneficial. Someone who has experienced homelessness, mental illness, and hepatitis C firsthand would understand the unique challenges and barriers we face and could offer valuable insights and support. However, I also recognize the importance of having a team of healthcare professionals.”—P8

“A patient who has been treated. Because you can advise more than the social worker. Because you’ve been there. You can explain to him all the steps to take. Usually that helps. Because normally, someone who is old and who is healed, if there is one who says… “Ah?” You can explain everything to him, from A to Z! Every step he has to take. For sure, you’re going to explain it to him.”—P9

While some acknowledged that a patient navigator with lived experience might offer a deeper understanding, the majority did not view it as essential for effective support. Many participants emphasized that instead, the patient navigator’s knowledge of HCV and its treatment were key.

“Well, of course, if it’s someone who has it, he’ll understand more, but someone who doesn’t have it, it’s maybe, sometimes … I’m still comfortable explaining that I have hepatitis C, as long as they know what it is and knows information on it … because it’s curable and because there’s a treatment.”—P6

Another participant suggested that, in some cases, individuals without lived experience might be more effective in ensuring engagement in HCV care than people with lived experience; instead, an essential quality was willingness to help.

“Well, not really, anyone can participate [as a patient navigator]. You don’t have to have a background in it to want to help, you know. Well, even if you’ve never used, even if you’re not a former drug user, if you haven’t had the disease, or if you’re not a nurse, actually, you make a better follow-up than them!”—P5

Other participants felt that a patient navigator with lived experience of incarceration could cause more harm than good, citing that they would struggle to trust them and their motives.

“It’s not an obligation [to have someone with a similar situation]. I wouldn’t feel comfortable with a guy from prison. Even in prison, they don’t understand sometimes that what they say can hurt somebody. But, you know, they don’t understand that it hurts me, because … some of them understand, some don’t understand as much.”—P10

“Definitely not someone who’s been to prison. You never know their motive.”—P8

“I don’t trust people in prison. I don’t want to rely on them.”—P9

Participants valued accurate information and professional support over shared personal experiences. Participants’ willingness to engage with patient navigators, regardless of their personal history of HCV and/or incarceration, highlights a broader acceptance of support. This suggests that while lived experience can be beneficial, individuals were primarily concerned about the patient navigator’s knowledge and competency in managing their health care needs.

## Discussion

This study explored the multi-level perceived barriers and facilitators to HCV care following release from prison using the SEM, as well as the perceived patient navigator services and characteristics best suited to address these barriers. Barriers to linkage to HCV care included competing priorities post-release (e.g., substance use, mental health issues, unstable housing), stigma (related to HCV, injection drug use, and incarceration), and lack of transportation to medical appointments. Facilitators included social support, established relationships with existing healthcare providers, prior cure with direct-acting antivirals, and HCV-related health literacy and knowledge. To overcome these barriers, patient navigator services that included pre-release discharge appointments and transportation and housing assistance were perceived to be crucial. Consistency, reliability, and empathy were considered important patient navigator characteristics while lived experiences of incarceration and/or HCV were not considered essential. These findings underscore the importance of patient navigation in improving linkage to HCV care post-release and may help inform future interventions.

Release from prison was associated with multiple stressors including homelessness, substance use, and self-isolation due to stigma of various forms. Other studies have also found that other priorities (e.g., employment and housing) often take precedence to linkage to health care following release (Crowley et al., 2019; Akiyama et al., 2020; Kamat et al., 2023). Future interventions could seek to improve HCV-related health literacy (knowledge and attitudes) (Mambro et al., 2024b), which participants expressed was an important facilitator to linkage to HCV care. Information that is both reliable and tailored to various levels of literacy was perceived to lead to increased motivation, ultimately empowering individuals to make informed decisions, critical to behaviour changes among people in prison (Zolotarova et al., 2023). In addition to health literacy, many participants highlighted that engagement in HCV care was part of a broader identity transformation, a process of overcoming their past and reshaping their lives. This finding aligns with another study (Donaldson et al., 2022), where health interventions, such as HCV treatment, were viewed as key to recovery and self-reinvention. That said, most people released from prison may not link to care without first ensuring that their primary and basic needs (e.g., housing, substance use treatment) are met, underscoring the importance of simultaneously addressing competing priorities at the time of release with any intervention that seeks to improve linkage to care. Participants attributed much of their success in accessing treatment to re-entry programs that provided the necessary stability and programming to engage in HCV care. Most emphasized the need for supportive housing options following community re-integrations. This will likely require individualized and integrated approaches to meet the needs of recently incarcerated individuals and require collaborations between health and long-term care, public health, housing, social assistance, and other community agencies (Alston et al., 2024; Boozary et al., 2024). Task shifting to the patient navigator for housing and community support services was an interesting but not unexpected finding in our study, underscoring that patient navigators may benefit from wholistic training that extends beyond pre-release discharge planning and transportation to include assistance with social services.

Interpersonal relationships were key facilitators to linkage to HCV care post-release. For example, participants expressed a preference to be linked to their existing health system, a finding that has been corroborated elsewhere (Akiyama et al., 2019). While only 10 % of participants had an existing healthcare provider, this finding suggests increased familiarity with the health care system may facilitate linkage to care. Supportive health care interactions build strong patient-provider relationships, which are essential for enhancing the overall quality of care and ensuring that patients remain engaged in care. Furthermore, social support from family or friends—that is, feeling that loved ones or friends are concerned with one’s wellbeing—was perceived to be an important facilitator to linkage to HCV care. Family support provides an additional layer of encouragement (beyond the patient navigator), reinforcing the importance of a supportive home environment in achieving successful health outcomes (Akiyama et al., 2019). In fact, studies have demonstrated that individuals are motivated to start HCV treatment as an act of reciprocity to their family and to improve inter-personal relationships (Western et al., 2005; Daly & Silver, 2008; Abdelwadoud et al., 2021). Where these relationships do not exist, patient navigators can help fill an important void.

The patient navigator, and the relationships fostered between the patient navigator and participants, were deemed to be critical for linkage to HCV care post-release. Participants expressed several qualities that patient navigators should embody to create a relationship built on trust, including being consistent and reliable, encouraging, and non-judgmental. In a recent study, cultural competency, good communication skills, and empathy were considered crucial characteristics for patient navigators to possess (Falade-Nwulia, 2022). Interestingly, “peers” were not perceived by most participants to be facilitators to linkage to HCV care, implying that lived or living experiences may not be critical for improving linkage to care following release from prison. A similar finding was reported in a recent study with peers in primary care settings, where shared health or social conditions were not found to be necessary to establish relationships of trust (Lessard et al., 2024). Instead, those who were able to “leverage their experiential knowledge, strengths, and abilities” were able to create the most meaningful relationships. These findings may challenge a recent recommendation made by WHO for the integration of peer navigators to support people from key populations to start viral hepatitis treatment and to remain in care (WHO, 2022b), where community-led services with peers have been shown to have a positive impact on HIV and viral hepatitis, with increased access, availability, and acceptability of services (Nguyen et al., 2019; Vannakit et al., 2020; Reza-Paul et al., 2019; Phanuphak et al., 2018; Onovo et al., 2021; Stardust et al., 2018). Rather, participants emphasized the importance of HCV knowledge and a genuine willingness to help others who were more disadvantaged. Our findings suggest that patient navigators without lived experience may foster equally meaningful relationships with patients and prior experiences with HCV or incarceration should not be considered a pre-requisite for successful patient navigation interventions.

A key patient navigator service identified in linkage to HCV care post-release was the pre-release discharge appointment. The patient navigator met with participants at least twice before their release, fostering a relationship prior to discharge that undoubtedly contributed to continuity of care post-release. In fact, many participants expressed that “familiarity” with the patient navigator post-release was directly due to their established relationships, which helped alleviate the emotional stress associated with navigating a complex health care system. Discharge planning has been found to improve linkage to HIV care post-release (Yanes-Lane et al., 2020) and future interventions should seek to routinely integrate this service to improve linkage to HCV care as well.

Our study, unlike others that have involved HCV patient navigation following release from carceral settings (Cabezas et al., 2021; Akiyama et al., 2019; Papaluca et al., 2022), is unique in that participants were offered transportation assistance (e.g., bus or subway passes, taxis) to their medical appointments by the patient navigator. Moreover, the patient navigator offered accompaniment to appointments if desired, which many felt was also crucial in linking to care. This active form of outreach and support by the patient navigator, including paid transportation services, was considered a critical facilitator to linkage to HCV care post-release. Furthermore, distance to the healthcare provider was an important consideration for most participants, with some preferring to wait for appointments with providers who were geographically closer than having expedited care further away. This preference for proximity has also been observed in other settings, such as community pharmacies for HCV care, where patients showed a willingness to wait for more familiar or convenient services (Radley et al., 2019). Our findings are also supported by a recent systematic review that found that transportation assistance facilitates linkage to HIV care post-release (Yanes-Lane et al., 2020), although our study is one of the first to demonstrate its importance in linkage to HCV care following release from prison. Accompaniment by a patient navigator helps facilitate continuity of care from prison to the community, which many participants expressed as being a time when many “fall through the cracks”. Until markedly improved collaborations between prisons and surrounding communities are established, patient navigators may help bridge this gap. Our findings point to the importance of integrating transportation assistance and patient accompaniment into all future interventions that seek to improve linkage to HCV care for people after release from prison, and support more decentralized and accessible HCV care through primary care.

Our study has limitations. First, all interviews were conducted at the time of linkage to HCV care by the patient navigator, meaning that social desirability bias was likely introduced, and personal experience may have influenced participants’ conceptualization of the patient navigator. Ideally, these interviews should have been performed by someone who was not a member of the prison-based multidisciplinary care team. That said, given the familiarity of the patient navigator to participants, there was the potential for a level of comfort during the interviews which may not have been achieved with an interviewer outside the multidisciplinary care team. Second, while interviews intentionally focused on the role of the patient navigator in the post-release period, individuals consented to participation in a larger intervention consisting of a multidisciplinary care model, meaning that the perceived views and attitudes of the patient navigator were difficult to disentangle from the overall intervention. Third, a more thorough understanding of post-release barriers to HCV care could have been achieved by interviewing individuals released from prison who were not linked to care. While this was our initial intention, these individuals were all lost to follow-up. Fourth, as all interviews were conducted in French, they required translation to English for analysis. In doing so, certain meanings and concepts may have been lost in translation. That said, the interviewers were fluent in both English and French and all transcripts were verified for accuracy. Fifth, our results may not be generalizable to women released from prison, whose priorities may differ from men. Sixth, as we collected minimal sociodemographic characteristics of participants, our findings may not be representative of the broader incarcerated population in Quebec or Canada that require linkage to HCV care post-release. Finally, while we interviewed only 10 participants, we did reach data saturation of themes.

## Conclusion

To our knowledge, this is the first study to characterize patient navigator services and characteristics to enhance linkage to HCV care among people released from prison and linked to care using patient navigation. Interventions that seek to improve linkage to HCV care for people following release from prison should consider patient navigator services that include pre-release discharge appointments and transportation and housing assistance, with care ideally provided by an existing healthcare provider if possible. While people experience many competing priorities post-release, ensuring a consistent, non-judgemental, and empathetic patient navigator, with or without lived experience of HCV and/or incarceration, may help overcome many barriers to linkage to HCV care post-release.

## Supplementary Material

Supplementary Materials

Supplementary material associated with this article can be found, in the online version, at doi:10.1016/j.drugpo.2024.104624.

## Figures and Tables

**Table 1:** The socio-ecological model levels and definitions, and example questions from the interview guide.

Socio-ecological model level	Classic definition	Example question(s) from the interview guide

1. Individual	Everything people are born with and how they influence and are influenced by the world around them (e.g., knowledge, substance use, mental health, unstable housing)	1.1. How do you access information on HCV and HCV care? To what extent has this information influenced you to seek HCV care? 1.2. Have you been treated for substance use? To what extent did this treatment influence you to seek care?
2. Interpersonal	Formal and informal social supports (e.g., family, friends, healthcare providers, peers)	2.1. Have you talked to your family, friends or peers about getting HCV care? 2.2. To what extent did these conversations influence you in gettisng HCV care post-release? 2.3. What services did the patient navigator provide that assisted in getting HCV care? 2.4. What characteristics should an “ideal” patient navigator possess to assist others in getting HCV care post-release?
3. Organizational	Relationships between public, private, and non-profit organizations and institutions (e.g., accessibility and coordination with public health care centres)	3.1. Did you experience any challenges in accessing health care following release from prison? 3.2. To what extent did these challenges influence your ability to seek HCV care post-release?
4. Community	The broad social setting in which relationships occur (e.g., community-based organizations, local outreach programs)	4.1. How have community resources impacted your ability to access HCV care after release? 4.2. What changes do you think are needed to community resources to better support individuals in similar situations?
5. Policy	Laws and policies that regulate and support health behaviours (e.g., local, provincial, federal)	5.1 What policies are needed to better support and facilitate linkage to HCV care after release?

**Table 2:** Baseline characteristics among study participants (*n* = 10).

Age; median (range)	54 (32–69)
Ethnicity	
White; *n* (%)	8 (80 %)
Indigenous (First Nations, Inuit, or Metis)	1 (10 %)
Other visible minority	1 (10 %)
Completed high school; *n* (%)	5 (50 %)
Age at first incarceration; median (range)	20 (18–35)
History of injection drug use; *n* (%)	9 (90 %)
Age at first use; median (range)	20 (16–51)
Newly diagnosed with HCV in prison; *n* (%)	2 (20 %)
Previously treated for HCV; *n* (%)	8 (80 %)
Known mental health diagnosis; *n* (%)	6 (60 %)
Existing primary care provider; *n* (%)	1 (10 %)
Injection drug use post-release; *n* (%)	1 (10 %)
Non-injection drug use post-release; *n* (%)	8 (80 %)
Unstable housing* post-release; *n* (%)	8 (80 %)

* Defined as living in a transition/halfway/safehouse or having no fixed address.

## Data Availability

Study data and materials may be shared following a request to the corresponding author.
